# Epidural Anesthesia and Continuous Epidural Analgesia in a Pediatric Patient With Pelizaeus-Merzbacher Disease: A Case Report

**DOI:** 10.7759/cureus.29983

**Published:** 2022-10-06

**Authors:** Toyoaki Maruta, Yumi Watanabe, Yukiko Nagata, Rina Kashino, Isao Tsuneyoshi

**Affiliations:** 1 Department of Anesthesiology, University of Miyazaki Hospital, Miyazaki, JPN

**Keywords:** analgesia, anesthesia, pelizaeus-merzbacher disease, pediatrics, epidural analgesia, epidural anesthesia, congenital hypomyelinating leukodystrophy

## Abstract

Pelizaeus-Merzbacher disease (PMD) is a genetic leukodystrophy, which is a progressive and degenerative central nervous system abnormality caused by dysmyelination. Because the incidence of PMD is extremely low, only a few case reports have been published regarding its anesthetic management. In particular, epidural anesthesia has only been reported in one case of general anesthesia combined with caudal anesthesia. We performed general anesthesia combined with epidural anesthesia for the soft-tissue release surgery for bilateral hip subluxation in a six-year-old male patient diagnosed with PMD. General anesthesia was induced with sevoflurane in nitrous oxide and oxygen. Rocuronium was administered to facilitate tracheal intubation. After intubation, general anesthesia was maintained with sevoflurane in the air and oxygen. An epidural catheter was placed from L3/4. For epidural anesthesia and analgesia, 1% mepivacaine was used as needed, and 2 ml/h of 0.2% ropivacaine was started one hour before the end of surgery. During surgery, only epidural analgesia was provided as postoperative analgesia, and the patient did not complain of pain after extubation. Anesthesia lasted three hours and 55 minutes. No significant hemodynamic or respiratory changes occurred. Postoperatively, the patient received continuous epidural analgesia and regular oral acetaminophen, and pain control was good. The epidural catheter was removed on the second postoperative day. The postoperative course was good, and the patient was transferred to a pediatric rehabilitation hospital on the fifth postoperative day. No adverse events occurred and no neurological deficits were observed during hospitalization. In conclusion, anesthesiologists should pay attention to the possibility of perioperative aspiration, spasticity, and seizure, even with mild PMD. Proper preoperative evaluations, intraoperative monitoring, and anesthetic techniques will ensure safe anesthesia for PMD patients. Although regional anesthesia in patients with pre-existing neurologic deficits is controversial, we were able to safely perform epidural anesthesia and postoperative continuous epidural analgesia in a pediatric patient with PMD.

## Introduction

Pelizaeus-Merzbacher disease (PMD) is the most frequent type of 11 recognized disorders of congenital leukodystrophy caused by the dysmyelination of the central nervous system [1,2]. It is characterized by nystagmus and head tremors in early infancy, and over time, mental retardation, spastic quadriplegia, dystonia, and cerebellar ataxia may also occur. The frequency in Europe and the United States is reported to be one in 200,000 to 500,000 live births and the number of patients in Japan is estimated to be about 200 [3]. Because the incidence of PMD is extremely low, only a few case reports have been published regarding its anesthetic management [4-8]. In particular, epidural anesthesia has only been reported in one case of general anesthesia combined with caudal anesthesia [5]. In this report, we describe a case of general anesthesia combined with epidural anesthesia and postoperative continuous epidural analgesia in a pediatric patient with PMD.

## Case presentation

A six-year-old male pediatric patient with a weight of 21 kg and a height of 97 cm was attending the hospital with PMD. He had nystagmus and developmental delay (holding his head up at four months, rolling over at six months, crawling at one year old, and no meaningful speech until six years old); however, he had not been medicated for PMD and experienced muscle weakness, seizures, and aspiration. Soft-tissue release surgery to treat the bilateral hip subluxation was scheduled.

**Figure 1:**
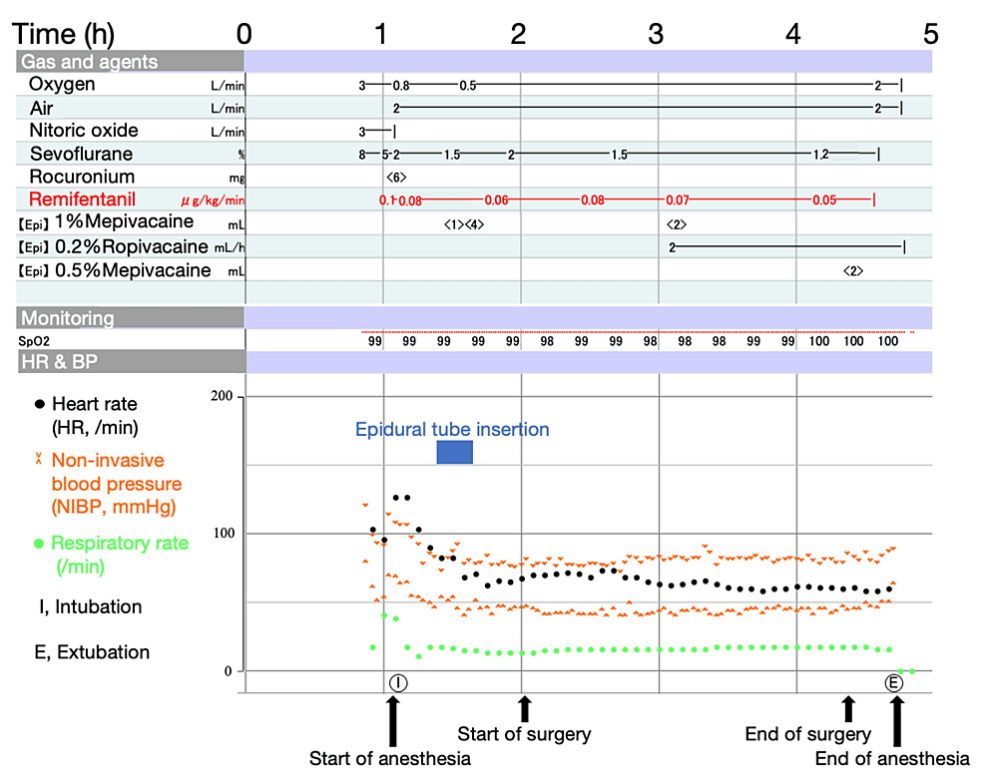
Anesthesia chart

Figure 1 shows the anesthesia chart. On his arrival in the operating room, standard anesthetic monitoring, including electrocardiography, pulse oximetry, noninvasive blood pressure, and end-tidal CO2, was initiated. General anesthesia was induced with sevoflurane in nitrous oxide and oxygen. About three minutes after the administration of 6 mg (0.3 mg/kg) of rocuronium, tracheal intubation was performed without complications such as airway trauma or hemodynamic instability. Sevoflurane (0.7 MAC) in air and oxygen and intravenous administration of remifentanil (0.05-0.1 μg/kg/min) were used to maintain general anesthesia. After intubation, he was placed in the left lateral position and an epidural catheter was placed. A Tuohy needle was inserted from L3/4 and loss of resistance was obtained at a depth of 1.5 cm. The epidural catheter was placed 5 cm to the cephalic side. For epidural anesthesia, 1% mepivacaine (1-4 ml) was used as needed, and 2 ml/h of 0.2% ropivacaine was started one hour before the end of surgery. During emergence from anesthesia, no unpredictable events were encountered. After extubation, the patient did not complain of pain with only epidural analgesia.

The operative time was two hours and 21 minutes, and anesthesia lasted three hours and 55 minutes and was completed without any events. No significant hemodynamic or respiratory changes occurred. He exhibited no signs of respiratory depression, and his muscle power almost completely recovered. Postoperatively, the patient received continuous epidural analgesia for 46 hours and regular oral acetaminophen, and pain control was good. The epidural catheter was removed on the second postoperative day. The postoperative course was good, and the patient was transferred to a pediatric rehabilitation hospital for postoperative rehabilitation on the fifth postoperative day. No adverse events occurred and no neurological deficits were observed during hospitalization.

## Discussion

There are no restrictions on the use of anesthetic agents and analgesics in general anesthesia in patients with PMD [8]. However, suxamethonium can cause unpredictable reactions such as hyperkalemia and should be avoided in rapid induction [4-8]. Considerations in anesthesia should include efforts to prevent complications associated with abnormal muscle tone due to spasticity, airway complications due to decreased pharyngeal muscle strength, aspiration, and convulsions. Rapid induction is essential when the risk of aspiration due to gastroesophageal reflux or decreased pharyngeal muscle tone is high. During emergence from general anesthesia, exacerbation of spasticity is the most common PMD-related complication and may persist after neuromuscular blockade has ceased; train of four (TOF) monitoring is useful to confirm recovery of neuromuscular function and detect spasticity [4]. Spasticity causes muscle contractions that may lead to respiratory failure, hypertension, dehydration, and rhabdomyolysis [4,8]. If exacerbation of spasticity is observed, benzodiazepines may be effective. To prevent convulsions, it is better to avoid high concentrations of anesthetics and hyperventilation [4]. Anesthesia and analgesia should be considered so as not to interfere with postoperative respiratory status, and regional anesthesia is useful to reduce opioid use [4,5].

Because of the concept of the “double-crush phenomenon” [9], which is classically defined as a phenomenon in which compression at one site of a peripheral nerve makes it more susceptible to damage proximal and/or distal to that site, epidural or spinal anesthesia use in patients with pre-existing central nervous system disorder is controversial. However, there is little definitive evidence to support or negate the use of regional anesthesia in patients with multiple sclerosis (MS), an acquired demyelinating disease of the central nervous system, for example [10]. Peripheral nerve blocks have not been conclusively shown to be harmful in MS, and thus cannot be considered an absolute contraindication [10,11]. In contrast, considering that demyelinated nerve fibers are sensitive to the toxicity of local anesthetics, epidural anesthesia and analgesia are considered safer than spinal anesthesia. However, it would be prudent in both peripheral nerve blocks and nerve trunk blocks to reduce the concentration and total dose of local anesthetics to the minimum necessary level. Thus, the use of regional anesthesia and analgesia in patients with pre-existing neuropathy should be carefully decided considering the potential risks and benefits. The postoperative risk of new or worsening neurological symptoms should be explained to the patient, because of exposure to multiple exacerbating factors, regardless of the anesthetic technique chosen.

## Conclusions

Anesthesiologists should pay attention to the possibility of perioperative aspiration, spasticity, and seizure, even with mild PMD. Proper preoperative evaluations, intraoperative monitoring, and anesthetic techniques will ensure safe anesthesia for PMD patients. Although the use of regional anesthesia in patients with pre-existing neurologic deficits is controversial, we perform epidural anesthesia and postoperative continuous epidural analgesia in a pediatric patient with PMD, and good anesthetic management and postoperative analgesia were achieved.

## References

[1] Inoue K (2019). Pelizaeus-Merzbacher disease: molecular and cellular pathologies and associated phenotypes. Adv Exp Med Biol.

[2] Osório MJ, Goldman SA (2018). Neurogenetics of Pelizaeus-Merzbacher disease. Handb Clin Neurol.

[3] Numata Y, Gotoh L, Iwaki A (2014). Epidemiological, clinical, and genetic landscapes of hypomyelinating leukodystrophies. J Neurol.

[4] Kim H, Lim C (2019). General anesthesia for an adolescent with Pelizaeus-Merzbacher disease - a case report. Anesth Pain Med.

[5] Kapoor R, Zavala AM, Van Meter A, Williams UU, Porche VH, Owusu-Agyemang P (2018). Anesthetic challenges and successful management of a child with Pelizaeus-Merzbacher disease using general and caudal anesthesia. J Anaesthesiol Clin Pharmacol.

[6] Patel R, Kahana M (2018). Anesthetic management of a pediatric patient with Pelizaeus-Merzbacher syndrome: a case report. A A Pract.

[7] Kamekura N, Nitta Y, Takuma S, Fujisawa T (2016). General anesthesia for a patient with Pelizaeus-Merzbacher disease. Anesth Prog.

[8] Tobias JD (1992). Anaesthetic considerations for the child with leukodystrophy. Can J Anaesth.

[9] Upton AR, McComas AJ (1973). The double crush in nerve-entrapment syndromes. Lancet.

[10] Kopp SL, Jacob AK, Hebl JR (2015). Regional anesthesia in patients with preexisting neurologic disease. Reg Anesth Pain Med.

[11] Hebl JR, Horlocker TT, Schroeder DR (2006). Neuraxial anesthesia and analgesia in patients with preexisting central nervous system disorders. Anesth Analg.

